# Brains, tools, innovation and biogeography in crows and ravens

**DOI:** 10.1186/1471-2148-12-72

**Published:** 2012-05-29

**Authors:** Knud A Jønsson, Pierre-Henri Fabre, Martin Irestedt

**Affiliations:** 1 Center for Macroecology Evolution and Climate at the Natural History Museum of Denmark, University of Copenhagen, Universitetsparken, 15, DK-2100, Copenhagen Ø, Denmark; 2 Molecular Systematics Laboratory, Swedish Museum of Natural History, P.O. Box 50007, SE-104 05, Stockholm, Sweden

**Keywords:** Biogeography, Brain, Cognition, Crow, Systematics, Tool use

## Abstract

**Background:**

Crows and ravens (Passeriformes: *Corvus*) are large-brained birds with enhanced cognitive abilities relative to other birds. They are among the few non-hominid organisms on Earth to be considered intelligent and well-known examples exist of several crow species having evolved innovative strategies and even use of tools in their search for food. The 40 *Corvus* species have also been successful dispersers and are distributed on most continents and in remote archipelagos.

**Results:**

This study presents the first molecular phylogeny including all species and a number of subspecies within the genus *Corvus*. We date the phylogeny and determine ancestral areas to investigate historical biogeographical patterns of the crows. Additionally, we use data on brain size and a large database on innovative behaviour and tool use to test whether brain size (*i*) explains innovative behaviour and success in applying tools when foraging and (*ii*) has some correlative role in the success of colonization of islands. Our results demonstrate that crows originated in the Palaearctic in the Miocene from where they dispersed to North America and the Caribbean, Africa and Australasia. We find that relative brain size alone does not explain tool use, innovative feeding strategies and dispersal success within crows.

**Conclusions:**

Our study supports monophyly of the genus *Corvus* and further demonstrates the direction and timing of colonization from the area of origin in the Palaearctic to other continents and archipelagos. The Caribbean was probably colonized from North America, although some North American ancestor may have gone extinct, and the Pacific was colonized multiple times from Asia and Australia. We did not find a correlation between relative brain size, tool use, innovative feeding strategies and dispersal success. Hence, we propose that all crows and ravens have relatively large brains compared to other birds and thus the potential to be innovative if conditions and circumstances are right.

## Background

Crows are large passerine birds that are considered intelligent because of their flexible behaviour, problem-solving abilities and social learning [1,2]. Several species show a number of fascinating innovations including tool use in their foraging, the best-known example being that of the New Caledonian crow (*C*. *moneduloides*) [3-5]. Such innovations in foraging are not only unique but are also expected to require increased cognitive abilities, which have been shown to be related to a brain size relatively larger than that of other birds [1,4,6]. Thus, the combination of opportunistic behaviour and intelligence should make corvids highly adaptable, competitive and potentially good colonizers of new environments [7,8].

The family Corvidae (crows, jays, magpies and allies) contains 117 species [9] distributed across most continents except Antarctica. Within the family, crows (genus *Corvus*) make up about one third of the species diversity (40 species) and they occur on all continents except South America and Antarctica as well as in remote archipelagos such as Hawaii, Micronesia and Melanesia [10]. The Corvidae is part of the core Corvoidea radiation that contains more than 750 species. Recent studies have argued that the core Corvoidea originated in an archipelago environment north of Australia in the late Oligocene/early Miocene and dispersed via the historically complex Indo-Pacific archipelagos to the rest of the world [11]. Thus, we may expect that some or all of the core Corvoidea’s member groups could have been preadapted for dispersal and colonization and in the case of *Corvus*, it can be expected that this combined with large brains and the increased associated cognitive abilities would make them ideal dispersers and colonizers across the planet [8].

Species-level systematics within *Corvus* has been based largely on morphological data [12] or very sparse sampling for molecular phylogenies e.g. [13-15] and even vocalizations have been used to infer phylogeny e.g. *C. enca* and *C. mellori*, [16,17]. A molecular phylogeny based on extensive taxon sampling is required to establish systematic relationships within *Corvus* so that questions pertaining to historical biogeography, brain size and the evolution of innovative foraging habits and tool use might be addressed. Additionally, a robust and densely sampled phylogeny will provide a framework for future work on plumage evolution and various aspects of macroecology and macroevolution.

In the present study, we present a molecular phylogeny including all extant crow species and a number of subspecies sometimes assigned species rank [10]. We use the phylogeny to assess systematic relationships and to elucidate historical biogeographical patterns by dating the phylogeny and estimating ancestral areas across the tree. Furthermore, taking into account the *Corvus* phylogeny, we test whether (*i*) brain size is correlated with the ability to disperse to and colonize islands and (*ii*) brain size correlates with innovative feeding behaviour and tool use within crows.

## Methods

### Taxon sampling and laboratory procedures

We sampled all forty extant species of *Corvus*[9] (Table 1). Where possible we included multiple individuals and, for widespread species, multiple subspecies (e.g. *Corvus encaCorvus macrorhynchosCorvus coronoides* and *Corvus frugilegus*). We also included some well-documented closely related genera to test for monophyly of *Corvus*: *GarrulusPicaNucifraga*[14]. *Lanius* was used to root the tree.

**Table 1:** List of taxa included in the study

**Species**	**Origin of sample**	**Voucher number**	**GAPDH**	**ODC**	**ND3**	**ND2**
*Corvus albicollis*	South Africa	FMNH 447947		JQ024104	JQ024061	JQ023991
*Corvus albus*	South Africa	FMNH 443790	JQ023921	JQ024103	JQ024060	JQ023990
*Corvus bennetti*	Australia	ANWC 33292	JQ023900	JQ024082	JQ024019	JQ023945
*Corvus bennetti*	Australia	ANWC 52018	JQ023901	JQ024083	JQ024020	JQ023946
*Corvus brachyrhynchos*	USA	UWBM 86268	JQ023920	JQ024102	JQ024039	JQ023966
Corvus capensis*	Abyssinia	FMNH 370464				JQ023977
*Corvus caurinus*	USA	UWBM 58841	JQ023915	JQ024097	JQ024034	JQ023961
*Corvus corax*	Denmark	ZMUC 131662	JQ023891	JQ024073		JQ023935
*Corvus corone cornix*	Denmark	ZMUC 143486	JQ023894	JQ024076	JQ024013	JQ023939
*Corvus corone corone*	Denmark	ZMUC 138386	JQ023892	JQ024074	JQ024010	JQ023936
*Corvus coronoides coronoides*	Australia, QLD	ANWC 32675	JQ023908	JQ024090	JQ024027	JQ023953
*Corvus coronoides coronoides*	Australia, NSW	ANWC 49539	JQ023911	JQ024093	JQ024030	JQ023956
*Corvus coronoides coronoides*	Australia, NSW	ANWC 29239	JQ023904	JQ024086	JQ024023	JQ023949
*Corvus coronoides coronoides*	Australia, ACT	ANWC 34200	JQ023910	JQ024092	JQ024029	JQ023955
*Corvus coronoides perplexus*	Australia, WA	ANWC 31774	JQ023906	JQ024088	JQ024025	JQ023951
*Corvus coronoides perplexus*	Australia, WA	ANWC 31706	JQ023905	JQ024087	JQ024024	JQ023950
*Corvus coronoides perplexus*	Australia, WA	ANWC 50365	JQ023902	JQ024084	JQ024021	JQ023947
*Corvus coronoides perplexus*	Australia, WA	ANWC 31869	JQ023907	JQ024089	JQ024026	JQ023952
*Corvus coronoides perplexus*	Australia, WA	ANWC 50476	JQ023912	JQ024094	JQ024031	JQ023957
Corvus crassirostris*	Ethiopia	NRM 551730			JQ024002	JQ023927
*Corvus cryptoleucus*	USA	UWBM 80762	JQ023917	JQ024099	JQ024036	JQ023963
*Corvus dauuricus*	Mongolia	UWBM 58041	JQ023913	JQ024095	JQ024032	JQ023958
Corvus edithae*	Kenya	FMNH 370461			JQ024056	JQ023986
Corvus enca celebensis*	Sulawesi	RMNH 60561			JQ024042	JQ023969
Corvus enca compilator*	Borneo	RMNH 60563			JQ024059	JQ023989
Corvus enca pusillus*	Palawan	RMNH 100023			JQ024043	JQ023970
Corvus florensis*	Flores	RMNH 85140			JQ024046	JQ023973
*Corvus frugilegus frugilegus*	Denmark	ZMUC 143511			JQ024011	JQ023937
Corvus frugilegus pastinator*	China	NRM 570731			JQ024068	JQ023999
Corvus fuscicapillus*	New Guinea	AMNH 300970			JQ024048	JQ023975
Corvus hawaiiensis*	Hawaii	AMNH 196263				JQ023982
Corvus imparatus*	Mexico	AMNH 706673				JQ023978
Corvus insularis	New Britain	AM 0.60592	JQ023888	JQ024070	JQ024007	JQ023932
Corvus jamaicensis*	Jamaica	AMNH 155238			JQ024052	JQ023981
Corvus kubaryi*	Micronesia	NRM 570711			JQ024003	JQ023928
Corvus leucognaphalus*	Hispaniola	NRM 570710			JQ024004	JQ023929
Corvus macrorhynchos japonensis*	Japan	NRM 570732			JQ024069	JQ024000
Corvus macrorhynchos levaillantii*	N. Siam	NRM 570733			JQ024067	JQ023998
*Corvus macrorhynchos mandschuricus*	Russia	UWBM 47167	JQ023918	JQ024100	JQ024037	JQ023964
Corvus macrorhynchos philippinus*	Philippines	ZMUC 104586			JQ024054	JQ023984
Corvus meeki*	Bougainville	AMNH 221033			JQ024058	JQ023988
*Corvus mellori*	Australia	ANWC 45128	JQ023895	JQ024077	JQ024014	JQ023940
*Corvus mellori*	Australia	ANWC 52403	JQ023903	JQ024085	JQ024022	JQ023948
*Corvus mellori*	Australia	ANWC 34099	JQ023909	JQ024091	JQ024028	JQ023954
Corvus minutus*	Cuba	AMNH 501484			JQ024051	JQ023980
*Corvus monedula*	Denmark	ZMUC 143533	JQ023893	JQ024075	JQ024012	JQ023938
Corvus moneduloides*	New Caledonia	FMNH 268468			JQ024040	JQ023967
Corvus nasicus*	Cuba	NRM 570734			JQ024066	JQ023997
*Corvus orru*	Australia	ANWC 32239	JQ023898	JQ024080	JQ024017	JQ023943
*Corvus orru*	Australia	ANWC 50885	JQ023899	JQ024081	JQ024018	JQ023944
*Corvus ossifragus*	USA	UWBM 86680	JQ023914	JQ024096	JQ024033	JQ023960
Corvus palmarum*	Hispaniola	FMNH 352731			JQ024050	JQ023979
*Corvus palmarum*	Hispaniola	AMNH DOT 16134	JQ023922	JQ024105		JQ023992
Corvus pectoralis*	China	AMNH 261595			JQ024053	JQ023983
Corvus pectoralis*	China	NRM 570709			JQ024005	JQ023930
Corvus rhipidurus*	Niger	FMNH370467			JQ024057	JQ023987
Corvus ruficollis*	Iran	FMNH284717			JQ024055	JQ023985
*Corvus sinaloae*	Mexico	UWBM 81200	JQ023916	JQ024098	JQ024035	JQ023962
*Corvus splendens*	Singapore	UWBM 83598	JQ023919	JQ024101	JQ024038	JQ023965
*Corvus tasmanicus boreus*	Australia	AM 0.70670	JQ023889	JQ024071	JQ024009	JQ023934
*Corvus tasmanicus boreus*	Australia	AM 0.70687	JQ023890	JQ024072	JQ024008	JQ023933
*Corvus tasmanicus tasmanicus*	Australia	ANWC 44920	JQ023896	JQ024078	JQ024015	JQ023941
*Corvus tasmanicus tasmanicus*	Australia	ANWC 45502	JQ023897	JQ024079	JQ024016	JQ023942
Corvus tristis*	New Guinea	NRM 543594			JQ024006	JQ023931
Corvus tristis*	New Guinea	RMNH 22732			JQ024049	JQ023976
Corvus typicus*	Sulawesi	RMNH 101686			JQ024045	JQ023972
Corvus unicolor*	Sulawesi	AMNH 673967			JQ024041	JQ023968
Corvus validus*	Halmahera	RMNH 140643			JQ024047	JQ023974
Corvus violaceus*	Seram	RMNH 140590			JQ024044	JQ023971
*Corvus woodfordi*	Solomon Islands	UWBM 63090				JQ023959
*Corvus woodfordi*	Solomon Islands	AMNH DOT6705	JQ023923	JQ024106	JQ024062	JQ023993
**Outgroups**						
*Nucifraga caryocatactes*	Sweden	ZMUC 138408	JQ023924	JQ024107	JQ024064	JQ023995
*Garrulus garrulus*	Denmark	ZMUC 136378			JQ024063	JQ023994
*Pica pica*	Denmark	ZMUC 144204	JQ023925	JQ024108	JQ024065	JQ023996
*Lanius collaris*	Cameroon/Tanzania	GenBank/ZMUC 138905	FJ357916	EU272112	JQ024001	JQ023926
*Dicrurus bracteatus/hottentottus*	New Guinea/Philippines	GenBank	EF052813	EU272113	GQ145422	GQ145384
*Sturnus vulgaris*	Sweden	GenBank	EF441231	EF441253	GU816823	DQ146346
*Menura*	Australia	GenBank	EF441220	EF441242	AY542313	AY542313
*Pitta angolensis*	Tanzania	GenBank	AY336596	DQ785940	GU816799	GU816827
*Acanthisitta*	New Zealand	GenBank	EU726202	EU726220	AY325307	AY325307

Acronyms are: **AM**, Australian Museum, Sydney, Australia; **AMNH**, American Museum of Natural History, USA; **ANWC**, Australian National Wildlife Collection, Canberra, Australia; **FMNH**, Field Museum of Natural History, Chicago, USA; **NRM**, Naturhistoriska Riksmuseet, Stockholm, Sweden; **RMNH**, Rijksmuseum van Natuurlijke Histoire, Leiden, Netherlands; **UWBM**, University of Washington, Burke Museum, Seattle, USA; **ZMUC**, Zoological Museum, University of Copenhagen, Denmark. Asterisks after taxon names indicate that sequences were obtained from toe-pads of old museum specimens.

Two nuclear gene regions, ornithine decarboxylase (ODC) introns 6 to 7 (chromosome 3), and glyceraldehyde-3-phosphodehydrogenase (GAPDH) intron-11 (chromosome 1), and two mitochondrial markers NADH dehydrogenase subunit 2 (ND2) and subunit 3 (ND3) were sequenced and used to estimate phylogenetic relationships. Primer pairs used for amplification were: ND2: Lmet [18]/H6312 [19]; ND3: ND3-L10755/ND3-H11151 [20]; ODC: OD6/OD8 [21], G3P13/G3P14b [22]. For the old museum specimens we only sequenced the mitochondrial genes. Corresponding laboratory procedures for study skins are detailed in Irestedt et al. [23]. Additional internal primers were designed for this study, ND3-corvR1: GTCAAATAGTAGAAACAGGATTGC; ND3-CorvF1: TTTTCAATTCGATTCTTCCTAGT; ND2-CorvR1: CTTGAACTAGAAAGTATTTGGTTGC; ND2-CorvF2:CCCCTAATCTCAAAATCTCACCA; ND2-CorvR2: CCTTGTAGGACTTCTGGGAATC; ND2-CorvF3: CTAGGACTAGTGCCATTTCACTT; ND2-CorvR3: AGATAGAGGAGAAGGCCATAATT; ND2-CorvF4: CTGAATAGGACTAAACCAAACACAA; ND2-CorvR4: AGTGTTAGTAGGAGGATTGTGCT; ND2-CorvF5: CCACACTAATAACTGCATGAACAAA; ND2-CorvR5: TGTGGGGTGGAAGTGTGATTGT; ND2-CorvF6: TCACTACTGGGCCTCTTCTTCTA. Purified PCR products were cycle-sequenced using the Big Dye terminator chemistry (ABI, Applied Biosystems) in both directions with the same primers used for PCR amplification and run on an automated AB 3100 DNA sequencer. Sequences were assembled with SeqMan II (DNASTAR). Positions where the nucleotide could not be determined with certainty were coded with the appropriate IUPAC code. GenBank accession numbers are provided in Table 1.

### Alignment and phylogenetic analyses

Sequence alignment was performed using MegAlign. The concatenated alignment consisted of 2346 base pairs (bp) and the lengths of the individual alignments were GAPDH: 299 bp, ODC intron-6 and 7: 611 bp, NADH dehydrogenase subunit 2: 1041 and NADH dehydrogenase subunit 3: 395 bp. Coding genes (ND2 and ND3) were checked for the presence of stop codons or insertion/deletion events that would have disrupted the reading frame. We used Bayesian inference [24,25], as implemented in MrBayes 3.1.2 [26,27] to estimate phylogenetic relationships. The most appropriate substitution models were determined with MrModeltest 2.0 [28], using the Akaike information criterion [29,30]. Bayesian analyses for the concatenated data set were performed allowing the different parameters (base frequencies, rate matrix or transition/transversion ratio, shape parameter, proportion of invariable sites) to vary between the six partitions (GAPDH, ODC, 1st, 2nd, 3 rd codon positions for mtDNA and tRNA), i.e. mixed-models analyses [27,28]. Two independent runs initiated from random starting trees were performed for each data set, and in all MrBayes analyses, the Markov Chain Monte Carlo (MCMC) was run using Metropolis-coupling, with one cold and three heated chains, for 10 million (individual analyses) to 20 million (combined analysis) iterations with trees sampled every 100 iterations. The number of iterations discarded before the chains had reached their apparent target distributions (i.e. the length of the “burn-in” period) was graphically estimated using AWTY [31,32] by monitoring the change in cumulative split frequencies, and by the loglikelihood values and posterior probabilities for splits and model parameters. We used GARLI 0.95 [33] to perform maximum likelihood analyses on the concatenated data set. Five independent analyses of 50 million generations were performed. Nodal support was evaluated with 100 nonparametric bootstrap pseudoreplications.

### Dating analyses

To estimate the relative divergence times within *Corvus*, we used beast v.1.6 [34-36] and assigned the best fitting model, as estimated by mrmodeltest 2.0 [28], to each of the four partitions. We assumed a Yule Speciation Process for the tree prior and an uncorrelated lognormal distribution for the molecular clock model [35,37]. We used default prior distributions for all other parameters and ran MC^3^ chains for 50 million generations. The program Tracer [38] was used to assess convergence diagnostics. To obtain absolute date estimates we calibrated the tree using secondary calibration points derived from Barker et al. [39] who used various approaches to date the all Passeriformes tree. Thus we used the age of Acanthisittidae versus other passerines at 76 ± 8 My SD (age within 95% confidence intervals = 62.8–89.2 My) and the split between *Menura noveahollandiae* and all other oscines 63 ± 2 My SD (confidence intervals = 59.7–66.3 My). In order to apply these calibration points, some additional taxa were included in the dating analyses (see Table 1). We also compared our age estimates with the classic mitochondrial 2% rule [40].

### Biogeographical analysis

We used Bayes-lagrange[41] to assess ancestral patterns within *Corvus*. In a Maximum-Likelihood biogeographical analysis [42,43] as implemented in the software lagrange [[42]], ancestral areas are optimized onto internal nodes. lagrange enables maximum likelihood estimation of the ancestral states (range inheritance scenarios) at speciation events by modelling transitions between discrete states (biogeographical ranges) along phylogenetic branches as a function of time. With the Bayes-lagrange approach it is possible to optimize on multiple trees whereby topological uncertainty is taken into account. We sampled 2000 trees (by thinning the chain stochastically) from the MCMC BEAST output, and ran lagrange on all of them. The frequency of ancestral areas for clades was then recorded and plotted as marginal distributions on the majority-rule consensus tree derived from the MCMC. The major advantage of the Bayes-Lagrange method is that the marginal distributions for the alternative ancestral areas at each node in the tree are the product of both the phylogenetic uncertainty in the rest of the tree and the uncertainty in the biogeographical reconstruction of the node of interest.

We assigned species distributions to one or more of nine geographical areas for the Bayes-lagrange analysis basing these on evidence of historical relationships of tectonic plates and terranes in the Indo-Pacific [44,45]: Nearctic, Caribbean, Palaearctic, Africa, Indomalaya (including the Philippines), Wallacea, Australo-papua and the Pacific. The analysis was carried out using the maxareas (= 2) option in lagrange. However, we also ran additional analyses exploring the importance of changing the maxareas (setting maxareas = 3 and 4).

### Brain size, tool use and innovation

Data on brain size, which are considered a good proxy for cognition and intelligence [46], and body mass, were taken from Mlikovsky [47] and Iwanuik & Nelson [48]. Although the data are drawn from two sources, the data have been converted to reflect inner brain case volumes and are therefore directly comparable. These two datasets together include brain size data for 29 *Corvus* species [47,48]. By comparing the brain sizes for those species that are represented in both datasets it is clear that most discrepancies between the two datasets are explained by the size of the bird individuals measured. Therefore we believe that the measurements from the two datasets can be analysed combined. For a few species information on body mass was lacking in which case we used data from the CRC Handbook [49]. We compared the data on body mass used in our analyses [47-49] with body mass data provided in Handbook of Birds of the World [10] and found the data to be in agreement. After ln transforming the data we regressed brain size against body mass for 30 out of 40 species of *Corvus*. We also ran separate analyses based on the two datasets from Mlikovsky [47] and Iwanuik & Nelson [48] to account for potential differences in measuring body mass and brain size. We compiled data on tool use and innovative foraging behaviour from studies by Lefebvre et al. [4], Overington et al. [50] and Bentley-Condit & Smith [51]. Additionally, we searched the Handbook of Australian, New Zealand and Antarctic birds [52] for data on Australian crows. Altogether we found that 10 out of 40 *Corvus* species have a documented record of using tools and that 17 out 40 species use innovative foraging strategies. We note that opinions differ on what it means to be a “real” tool user and that some species are only known to use tools in captivity (*Corvus frugilegus*). However, this only underscores the high plasticity of this behaviour among *Corvus* species.

To investigate whether tool use, innovative foraging strategy and colonization of islands was associated with relative larger brain size across *Corvus* species, we ran a phylogenetic generalized least squares model (PGLS) in a phylogenetic framework using R version 2.10.1 [53] and the caper R package [54,55] as well as the ape package [56]. This statistical approach fits a linear model, taking into account phylogenetic non-independence. We tested the correlation of ln transformed brain size and ln transformed body mass as explanatory variables, with potential effect of tool use, innovation or island/continent distribution.

## Results

### Molecular phylogenetics and dating

**Figure 1:**
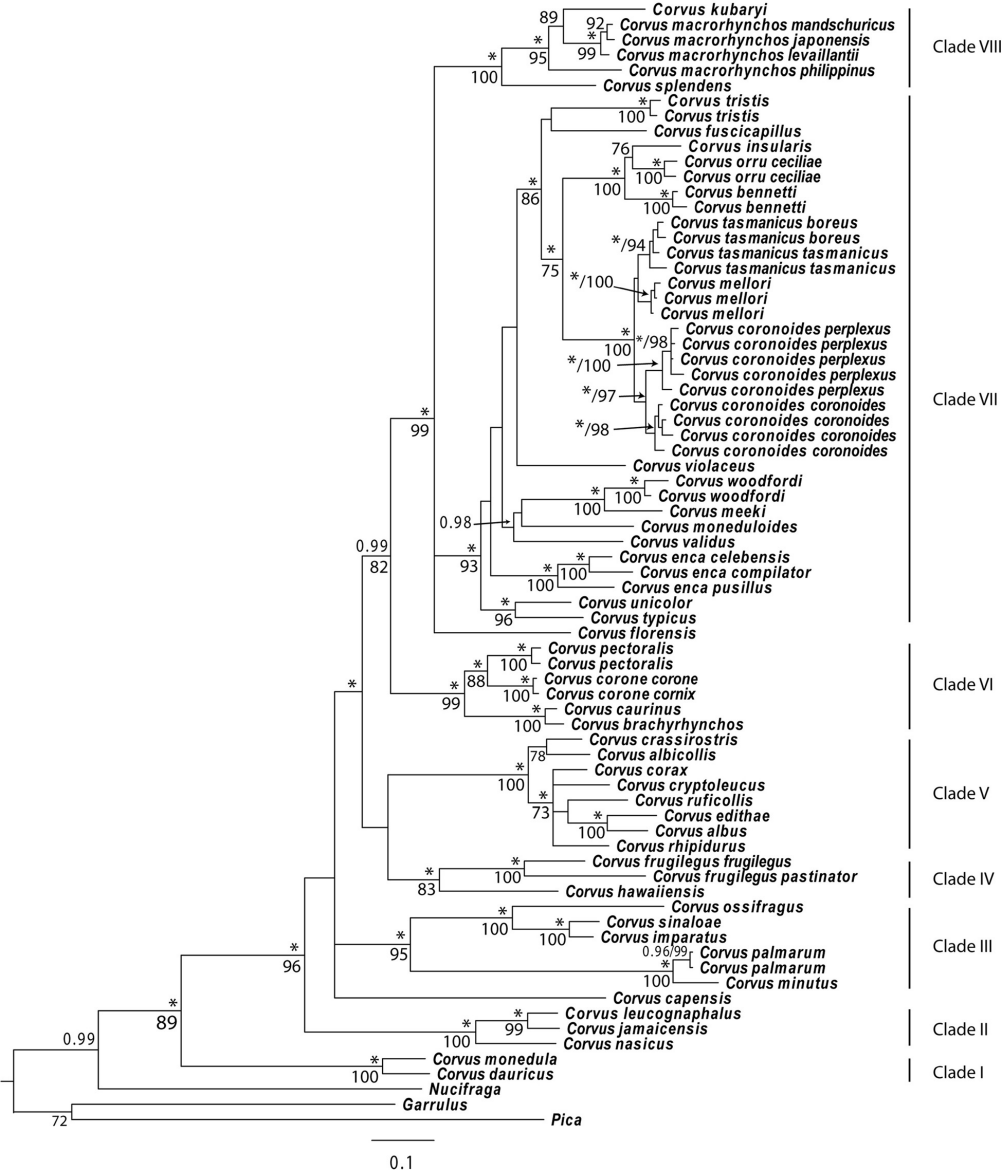
** The 50% majority-rule consensus tree of the *Corvus *obtained from the Bayesian analysis of the combined dataset (GAPDH, ODC, ND2 and ND3).** Above the branch is the posterior probability (only values above 0.95 are shown, asterisks indicate 1.00 posterior probabilities). Below the branch is the maximum likelihood bootstrap value (only values above 70% are shown) from 100 pseudoreplicates. Clades I-VIII are discussed in the text.

Model based analyses performed on the concatenated dataset (six partitions: GAPDH, ODC, 1st, 2nd, 3 rd codon positions for mtDNA and tRNA; maximum likelihood (ML): –ln 16528.5601, Bayesian inference (BI) harmonic mean: –ln 15872.64) yielded a 50% majority-rule consensus tree (BI) that was topologically congruent with the Maximum Likelihood tree (Figure 1), (for well-supported nodes receiving posterior probabilities >0.95 or bootstrap values >70%). Scores of the best likelihood trees were within 0.05 likelihood units of the best tree recovered in each of the other four garli runs, suggesting that the five runs had converged.

We find that the genus *Corvus* is monophyletic and furthermore recovered eight well-supported sub-clades that contain members more or less restricted to biogeographical regions. The basal members (Clades I-III) are distributed across the Holarctic region, the Caribbean and Africa. The Caribbean members are found in two well-supported separate clades (Clades II and III) but resolution between the clades and *Corvus capensis* remain unresolved. Clade IV consists of the Eurasian *Corvus frugilegus* and *Corvus hawaiiensis*. The western and eastern subspecies of *C*. *frugilegus* represent a deep split in concordance with a previous study on this species complex [15]. Clade V consists of all the African *Corvus* species (except *C*. *capensis*) and the Holarctic *C*. *corax*. Clade VI consists of Holarctic species that are separated in two distinct well-supported clades, one clade of Neararctic species (*C*. *caurinus* and *C*. *brachyrhynchos*) and one clade of Palaearctic species (*C*. *corone* and *C*. *pectoralis*). Clade VII contains all the Australo-Papuan and Wallacean taxa except the Wallacean *Corvus florensis*. The latter species remains unresolved relative to Clades VII and VIII. Within Clade VII we also find some Pacific taxa. One subclade within Clade VII contains all Australian taxa and we also recover a well-supported Australo-Papuan clade. The relationships of the Wallacean species, however, remain unresolved at the base of Clade VII except that there is good support for a sister relationship between *C*. *unicolor* and *C*. *typicus*. Clade VIII includes the widespread *C*. *macrorhynchos*, the Indo-Malayan *C*. *splendens* and the Micronesian *C*. *kubaryi*.

Our BEAST dating analysis supports a mid-Miocene origin of *Corvus* dating to around 17.5 Mya (age within 95% HPD confidence intervals = 14.05–21.19 My). Our chronogram was consistent with the “2% rule” (uncorrected pairwise distances) for the rate of mitochondrial DNA sequence divergence per million years for young nodes (Pliocene to present) but suggested somewhat younger diversification times than those that BEAST determined as of Miocene age, which could be expected due to saturation in the mitochondrial genes [57]. According to the 2% rule the origin of *Corvus* dates to about 11 Mya. Knowledge of a *Corvus* fossil from North America dating back to the late Miocene [58] does not add much further insight because the author was unable to assign a systematic position. However, assuming that the fossil is closely related to the other North American taxa it supports our age estimates based on secondary calibration points.

### Biogeographical analysis

**Figure 2:**
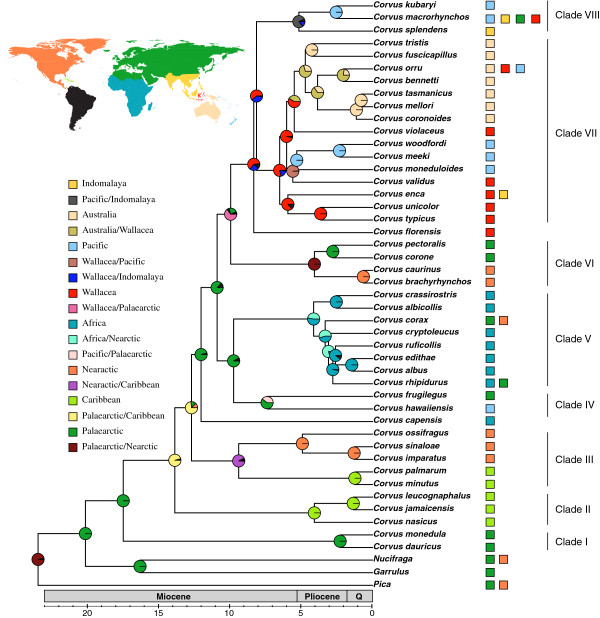
** A summary of the BAYES-LAGRANGE ancestral area analysis for the genus *Corvus. ***The tree is a chronogram (pruned to include one individual per species) based on the BEAST dating analysis of a combined data set of mitochondrial (ND2 and ND3) and nuclear (GAPDH and ODC) DNA sequences. Pie charts at internal nodes indicate the probability of a given area of origin. The inset map indicates the regions demarcated for the ancestral area analyses and colours to the right of the taxon names indicate present distributions (Nearctic, Palaeearctic, Caribbean, Africa, Indomalaya, Wallacea, Australo-Papua and Pacific) and thus coding for the ancestral area analyses. Black parts of the pie charts indicate a mixture of other areas.

The Bayes-lagrange analysis (Figure 2) finds the origin of *Corvus* and its closest relatives (*Pica*, *Nucifraga*, *Garrulus*) to be within the Holarctic region. The origin of *Corvus*, however, is Palaearctic, although some deep branches lead to taxa distributed in North America and the Caribbean. Colonization of Africa took place in the Pliocene, and colonization of Wallacea took place in the late Miocene and led to further colonization of Australo-Papua around 5 Mya. We find evidence for four colonization events of the Pacific from Asia and Australia. The Caribbean was colonized twice. One Caribbean clade is sister to a clade of North American taxa (Clade III) indicative of colonization from there. However, the ancestral area analysis postulates a Palaearctic/Caribbean origin for the other Caribbean clade (II).

### Brain size and tool use

**Figure 3:**
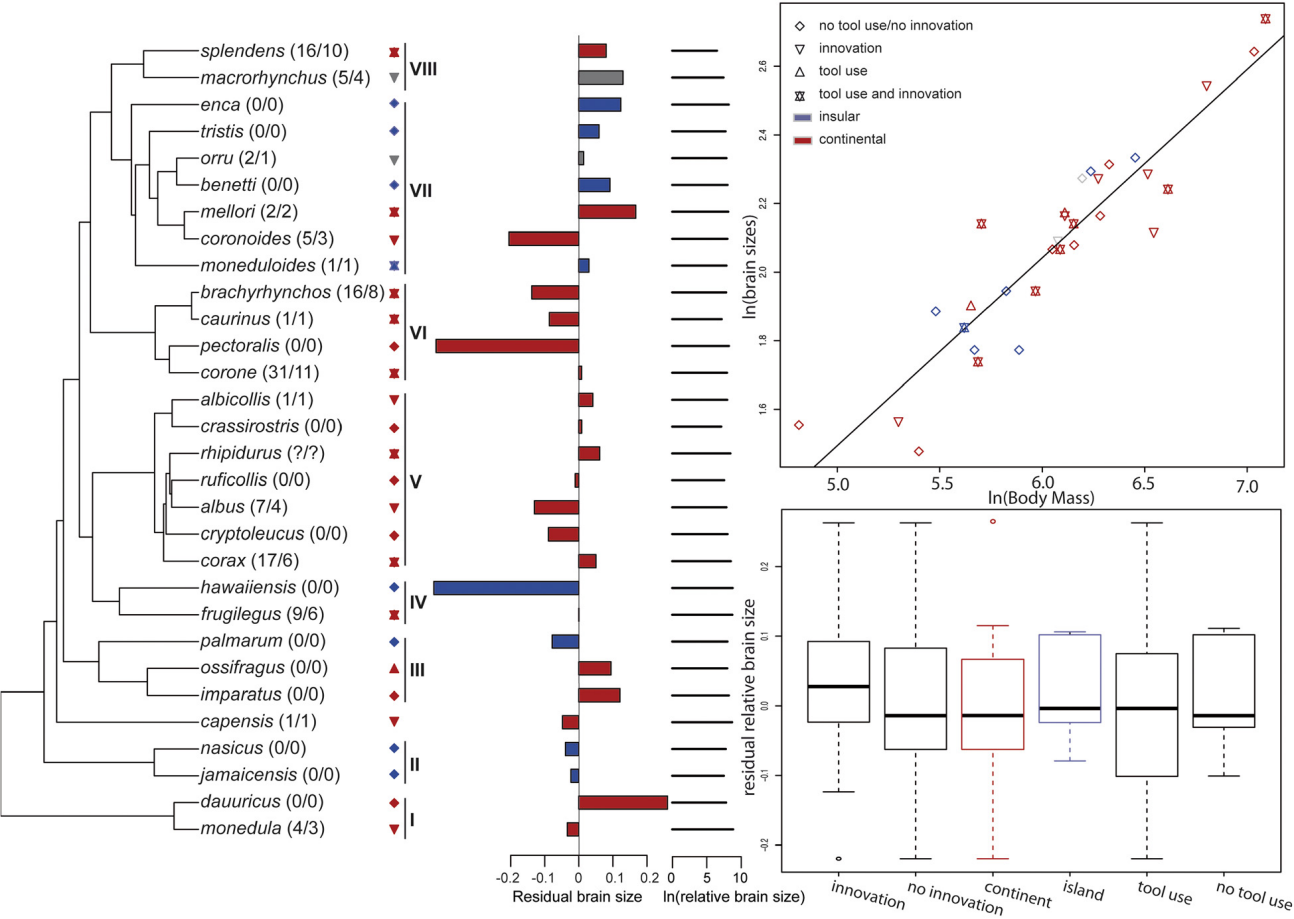
** A) Phylogeny showing the taxa used in the comparative brain size analyses.** Numbers in parentheses indicate the number of innovations followed by the diversity of innovations. Symbols indicate whether the taxon applies tools (upwards pointing triangle), innovative strategies (downwards pointing triangle) or both (star combining the two triangles) in its search for food. Distributions are indicated for islands (blue), continents (red) or both (grey). Island taxa are indicated in blue, continental taxa in red and combinations in grey. Residual brain size and relative brain size for the taxa are indicated to the right of the phylogeny **B)** Linear regression between brain and body mass. **C)** Box-plot displaying the difference (median, 25% and 75% percentiles and sample minimum and maximum) in relative brain size between *Corvus* species that use tools/no tools, *Corvus* species that apply innovation/no innovation and *Corvus* species that occur on islands/continents. Relative brain size represents residual values obtained from a linear regression between ln-transformed brain size and ln-transformed body mass.

We find a highly significant correlation between body mass and brain size in *Corvus* (P < 0.001 Figure 3) both for the combined dataset and when analyzing the individual datasets from Mlikovsky [47] and Iwanuik & Nelson [48]. This suggests that there are no significant differences in relative brain size between large and small *Corvus* species. Our analysis (Phylogenetic Generalized Least Squares) taking covariance between taxa into account, finds no correlation between brain size, tool use (P = 0.67) and innovative behaviour (P = 0.69), and no correlation between brain size and the ability to colonize islands (P = 0.46) (Figure 3C). Seemingly, all members of *Corvus* have the same relative brain size and species of all sizes have innovative foraging strategies/use tools and have been able to colonize islands.

## Discussion

### Systematics and biogeography

The early history of the classification of the family Corvidae has been summarized by Goodwin [12] but is restricted to morphology. We present the first complete molecular species level phylogeny for the crows and ravens (*Corvus* spp) including several subspecies of widespread species (Figure 1). Most notably, we demonstrate that what is currently classified as the Australian Raven *C. coronoides*, comprises two clades, one in the west (*C*. *c. perplexus*) and one in the east (*C*. *c*. *coronoides*). They are geographically isolated from each other only by approximately 100 km of apparently unsuitable habitat across the continent’s south coast at the Great Australian Bight. We propose that these two taxa be elevated to species rank. In contrast, populations of *C*. *tasmanicus*, geographically isolated from each other by >500 km, were not reciprocally monophyletic. Currently recognized as two subspecies, their isolation and divergence is presumably very recent. In accordance with a previous study including samples from throughout the Palaearctic, we show that *C*. *frugilegus* may also represent two distinct species, one in the western Palaearctic and one in the eastern Palaearctic [15]. *C. macrorhynchos* is found to be paraphyletic such that the Philippine *C*. *macrorhynchos philippinus* is sister to a clade comprising all other *C. macrorhyncos*, which occur in East Asia, and *C. kubaryi*. However, denser taxon sampling for *C*. *macrorhynchos* and population sampling for other widespread species (e.g. *Corvus enca* and *Corvus orru*) is needed to properly revise taxonomic issues at species and subspecies levels.

Our dating analysis suggests that the radiation of *Corvus* began in the mid-Miocene and our ancestral area analysis indicates a Palaearctic origin (Figure 2). This is consistent with the two most basal members, *Corvus monedula* and *Corvus dauuricus* (Clade I) being Eurasian and with the closest extant relatives of *Corvus* distributed in Eurasia and North America (*Pica* and *Nucifraga*). This results in a signature of a Holarctic origin for *Corvus* and its closest relatives. We infer that two clades independently colonized the Caribbean islands in the late Miocene (Clades II and III). One Caribbean clade (II) has a long branch that leads to *C*. *leucognaphalusC*. *jamaicensis* and *C*. *nasicus* and our analyses suggest a Palaearctic/Caribbean origin. This could be interpreted as evidence for long distance ocean dispersal similar to that inferred in other passerine bird groups that have crossed the Atlantic e.g. *Turdus*[59]. Two alternative interpretations of the Caribbean having been colonized from North America are possible (*i*) extinction of a North American ancestor, (ii) an ancestral form was widely distributed in the Holarctic (like *Corvus corax*) and gave rise to independent colonizations to the Caribbean followed by isolation of the North American population and a second colonization to the Caribbean (Clade III). The other Caribbean species (*C*. *palmarum* and *C*. *minutus*; Clade III) are sister to three North American species (*C*. *ossifragusC*. *sinaloae* and *C*. *imparatus*). This provides evidence for colonization by *C*. *palmarum* and *C*. *minutus* of the Caribbean from North America.

Relationships of an African species, *C. capensis*, were difficult to ascertain but it seems to represent a single Miocene colonization of Africa from the Palaearctic. *C. capensis* does not seem closely related to members of Clade V, which includes all other taxa that have colonized Africa and radiated within the continent. Clade VII of Indo-Pacific species has sequentially colonized Southeast Asia, Wallacea, Australo-Papua and the Pacific islands. However, the Hawaiian crow (*C*. *hawaiiensis*) is not a member of this clade, instead it clusters with the Palaearctic *C*. *frugilegus* (Clade IV) and so we infer it to have colonized Hawaii from East Asia. This is unexpected because the Hawaiian biota generally evolved through colonization from America whereas that of the rest of the Pacific was mostly colonized from Asia and Australo-Papua [60]. For the *Corvus* radiation, Asia has been the main source area for colonization of the Pacific as opposed to Australo-Papua, which is only the source area for one out of four Pacific lineages. Overall, the ancestral area analysis provides a rather clear pattern of separate colonizations of all continents except South America from the Palaearctic/(Nearctic).

## Brain size, tool use and colonization

Tool use is rare in the animal kingdom and is considered restricted to primates [61], Cetaceans [62], and some birds (e.g. Psittaciformes and Passeriformes) [1]. By far the most well-known tool using bird is the New Caledonian crow (*Corvus moneduloides*) and several studies have demonstrated this island endemic crow’s ingenious abilities to use sticks to probe for larvae [5,63]. It is well established in the literature that cognitive abilities correlate with larger relative brain size, and that the family Corvidae have unusually large brains compared to other birds [64,65]. Particularly, the New Caledonian crow’s ability to use tools has been explained by its extraordinary large brain [6]. However, in the study by Cnotka et al. [6] phylogenetic relationships among crows were unknown and they were therefore unable to make appropriate phylogenetic corrections.

Several members of the genus *Corvus* use a variety of natural tools or advanced innovative strategies when foraging [summarized in 4]. Until now, innovative feeding techniques have been reported for 17 of the 40 species of *Corvus* and tool use for 10 of the 40 *Corvus* species ( Additional file 1: Table S1). There is only one tool using species (*Corvus ossifragus*) that is not known to have foraging innovations and it should be noted that tool-use is usually seen as species typical and often hard-wired as evidenced by experiments on young woodpecker finches [66] and young New Caledonia crows [67], both of which “know” about tools from the start without having to learn about them.

Our analyses on body mass and brain size demonstrate that there is a significant correlation between the two variables, meaning that all crows have the same large relative brain size (similar body mass/brain size ratio). However, our comparative analyses on brain size and innovative feeding/tool use strategies within *Corvus*, corrected for phylogenetic relationships, reveal no correlation between the variables. This could be interpreted in two ways. Either brain size has little to do with innovative foraging strategies/tool use and thus other factors are more important in determining whether or not crows use tools or innovative feeding strategies. Alternatively, all crow species have large brains relative to other birds and thus have the potential to use tools or other innovative feeding strategies. Given that a number of studies have already demonstrated a link between cognitive abilities and brain size in both birds [3,4] and mammals [68,69] and that it is well-established that corvids have larger brains than many other birds [1,4], it seems the most likely hypothesis that all crows have the potential to develop innovative foraging strategies and to use tools in their search for food and there could be many reasons why this potential is only realised in some species across the *Corvus* tree. However, it has also been argued that total brain size may not be the ideal proxy for cognition and that measures should be taken to explore particular brain regions to explain innovation and tool-use [70]. We do not consider this study the final word on the topic, but merely a first attempt to combine phylogeny with functional traits associated with cognition and innovation in crows.

The most persistent hypothesis of large brains and corresponding enhanced cognition is that they evolved as an adaptation to handle novel or altered environmental conditions [71]. Island environments may prove particularly challenging as they, depending on the size and nature of the island, may provide fewer available niches, inferior access to food and new unknown dangers. On the other hand, a new island colonizer, could also find itself in an environment free of closely related competitors and free of inhibitors leading to occupancy of a wider range of habitats – ecological release [72,73]. A combination of these two extreme scenarios, however, could to some extent counteract each other, which may explain the lack of correlation between relative brain size and island colonisations in crows and ravens.

## Conclusion

The analyses based on molecular sequence data from all recognized crow and raven species (genus: *Corvus*) demonstrate that the genus is monophyletic and that it originated in the Palaearctic in the Miocene. From the centre of origin crows dispersed to North America and the Caribbean, to Africa and to Australasia, with several independent colonizations of remote Pacific islands. Our analysis comparing brain size and colonization of islands within *Corvus* found no correlation and we therefore conclude that colonization of islands by crows cannot be explained by brain size. We did not find a correlation between brain size, tool use and innovative foraging strategies as otherwise suggested by other studies e.g. [6,8]. Thus, there seems no reason to believe that brain size alone has any influence on tool use, innovative foraging stragegies and colonization ability within the crow lineages. Rather it would appear that large brains had already evolved in the ancestor of crows, leading to a generally high cognitive ability to deal with new challenges for crows and other corvid lineages.

## Competing interests

The authors have no competing interests.

## Authors’ contributions

KAJ designed the study, carried out the lab work, performed the phylogenetic analyses, and drafted the manuscripts. PHF carried out additional comparative analyses. MI assisted with lab work. All authors read, commented upon and approved the manuscript.

## Supplementary Material

Additional file 1 **Table S1.** List of taxa used in the comparative analyses Distribution is either insular or continental. Body mass (D) according to Dunning body mass (I) according to Iwanuik & Nelson and body mass (M) according to Mlikovsky. Brain size according to Mlikovsky and Iwanuik & Nelson. Feeding innovation according to Lefebvre et al. Bentley-Condit & Smith and Higgins et al. Feeding innovations according to Lefebvre et al. Bentley-Condit & Smith and Overington et al. [4,7,47-51]. Click here for file
