# Supplementary material for: Brains, tools, innovation and biogeography in crows and ravens
Source: BMC Evol Biol. 2012 May 29;12:72. doi: 10.1186/1471-2148-12-72 (PMC3480872; doi:10.1186/1471-2148-12-72)
Supplement: Additional file 1 — Table S1. List of taxa used in the comparative analyses Distribution is either insular or continental. Body mass (D) according to Dunning body mass (I) according to Iwanuik & Nelson and body mass (M) according to Mlikovsky. Brain size according to Mlikovsky and Iwanuik & Nelson. Feeding innovation according to Lefebvre et al. Bentley-Condit & Smith and Higgins et al. Feeding innovations according to Lefebvre et al. Bentley-Condit & Smith and Overington et al. [4,7,47-51]. [file 1471-2148-12-72-S1.doc]

**SI Table 1. List of taxa used in the comparative analyses Distribution is either insular or continental. Body mass (D) according to Dunning [1], body mass (I) according to Iwanuik & Nelson [2] and body mass (M) according to Mlikovsky [3]. Brain size according to Mlikovsky [3] and Iwanuik & Nelson [2]. Feeding innovation according to Lefebvre *et al*. [4], Bentley-Condit & Smith [5] and Higgins et al. [6]. Feeding innovations according to Lefebvre *et al*. [4], Bentley-Condit & Smith [5] and Overington et al. [7].**

| **Species** | **Distribution** | **body mass (D)** | **body mass (M)** | **body mass (I)** | **Brain size (M)** | **Brain size (I)** | **True tool use** | **Feeding innovations** |
| --- | --- | --- | --- | --- | --- | --- | --- | --- |
| *Corvus albicollis* | Continental |  | 900 |  | 12.7 |  |  | x |
| *Corvus albus* | Continental |  | 530 | 584.1 | 9.7 | 8.75 |  | x |
| *Corvus bennetti* | Continental |  | 430 | 379 | 7.9 | 6.44 |  |  |
| *Corvus brachyrhynchos* | Continental |  | 450 | 438.5 | 8.7 | 7.17 | x | x |
| *Corvus capensis* | Continental |  | 695 |  | 8.3 |  |  | x |
| *Corvus caurinus* | Continental |  | 390 | 384 | 7 | 7.43 | x | x |
| *Corvus corax* | Continental |  | 1200 | 1051.9 | 15.4 | 14.45 | x | x |
| *Corvus corone* | Continental |  | 470 | 536.5 | 8.5 | 8.51 | x | x |
| *Corvus coronoides* | Continental |  |  | 675 |  | 9.83 |  | x |
| *Corvus crassirostris* | Continental | 1135 |  |  | 14 |  |  |  |
| *Corvus cryptoleucus* | Continental |  | 535 | 534 | 8.7 | 8.95 |  |  |
| *Corvus dauuricus* | Continental |  |  | 123 |  | 4.75 |  |  |
| *Corvus enca* | Insular |  | 240 |  | 6.6 |  |  |  |
| *Corvus frugilegus* | Continental |  | 440 | 488 | 7.9 | 7.61 | x | x |
| *Corvus hawaiiensis* | Insular | 511 |  |  | 9.9 |  |  |  |
| *Corvus imparatus* | Continental | 221 |  |  | 4.4 |  |  |  |
| *Corvus jamaicensis* | Islands | 338 |  |  | 7 |  |  |  |
| *Corvus macrorhynchos* | Insular/Continental |  | 490 |  | 9.7 |  |  | x |
| *Corvus mellori* | Continental |  |  | 300 |  | 8.5 | x | x |
| *Corvus monedula* | Continental |  | 200 | 246 | 4.8 | 5.15 |  | x |
| *Corvus moneduloides* | Insular |  | 275 | 267.5 | 6.3 | 7.23 | x | x |
| *Corvus nasicus* | Insular |  | 360 |  | 5.9 |  |  |  |
| *Corvus orru* | Insular/Continental |  | 435 | 522.2 | 8.1 | 8.91 |  | x |
| *Corvus ossifragus* | Continental |  | 285 | 285 | 6.7 | 5.99 | x |  |
| *Corvus palmarum* | Insular |  | 290 |  | 5.9 |  |  |  |
| *Corvus pectoralis* | Continental | 471 |  |  | 8 |  |  |  |
| *Corvus rhipidurus* | Continental |  | 745 |  | 9.4 |  | x | x |
| *Corvus ruficollis* | Continental | 559 |  |  | 10.1 |  |  |  |
| *Corvus splendens* | Continental |  | 295 |  | 5.7 |  | x | x |
| *Corvus tristis* | Insular |  | 635 |  | 10.3 |  |  |  |
| **Data deficient taxa** |  |  |  |  |  |  |  |  |
| *Corvus florensis* | Insular |  |  |  |  |  |  |  |
| *Corvus fuscicapillus* | Insular |  |  |  |  |  |  |  |
| *Corvus kubaryi* | Insular | 248 |  |  |  |  |  |  |
| *Corvus leucognaphalus* | Insular |  |  |  | 8.7 |  |  |  |
| *Corvus sinaloae* | Continental | 244 |  |  |  |  |  |  |
| *Corvus tasmanicus* | Continental | 678 |  |  |  |  |  | x |
| *Corvus typicus* | Insular |  |  |  |  |  |  |  |
| *Corvus unicolor* | Insular |  |  |  |  |  |  |  |
| *Corvus validus* | Insular |  |  |  | 8.7 |  |  |  |
| *Corvus woodfordi* | Insular | 467 |  |  |  |  |  |  |

**SI references**

1. Dunning JB Jr: *The CRC handbook of avian body masses*. CRC Press, Boca Raton, FL, USA; 1993.

2. Iwaniuk AN, Nelson JE: **Developmental differences are correlated with relative brain size in birds: a comparative analysis.** *Can J Zool* 2003, **81**:1913-1928.

3. Mlikovsky J: **Brain size and forearmen magnum area in crows and allies (Aves: Corvidae).** *Acta Soc Zool Bohem* 2003, **67:**203-211.

4. Lefebvre L, Nicolakakis N, Boire D: **Tools and brains in birds.** *Behaviour* 2002, **139**:939-973.

5. Bentley-Condit VK, Smith EO: **Animal tool use: current definitions and an updated comprehensive catalog.** *Behaviour* 2010, **147:**185-221.

6. Higgins PJ, Peter JM, Cowling SJ: *Handbook of the Australian, New Zealand and Antarctic birds. Vol 7: Boatbills to starlings*. Melbourne, Australia: Oxford University Press; 2006.

7. Overington SE, Morand-Ferron J, Boogert NJ, Lefebvre L: **Technical innovations drive the relationship between innovativeness and residual brain size in birds.** *Animal Behav* 2009, **78:**1001-1010
